# Objective Assessment of Physical Activity at Home Using a Novel Floor-Vibration Monitoring System: Validation and Comparison With Wearable Activity Trackers and Indirect Calorimetry Measurements

**DOI:** 10.2196/51874

**Published:** 2024-04-25

**Authors:** Yuki Nakajima, Asami Kitayama, Yuji Ohta, Nobuhisa Motooka, Mayumi Kuno-Mizumura, Motohiko Miyachi, Shigeho Tanaka, Kazuko Ishikawa-Takata, Julien Tripette

**Affiliations:** 1 Department of Human-Environmental Sciences Ochanomizu University Bunkyo Japan; 2 Department of Performing Arts Ochanomizu University Bunkyo Japan; 3 Faculty of Sport Sciences Waseda University Tokorozawa Japan; 4 National Institute of Health and Nutrition National Institutes of Biomedical Innovation, Health and Nutrition Settsu Japan; 5 Faculty of Nutrition Kagawa Nutrition University Sakado Japan; 6 Faculty of Applied Biosciences Tokyo University of Agriculture Setagaya Japan; 7 Center for Interdisciplinary AI and Data Science Ochanomizu University Bunkyo Japan

**Keywords:** smart home system, physical behavior, physical activity, activity tracker, floor vibration, housework-related activity, home-based activity, mobile phone

## Abstract

**Background:**

The self-monitoring of physical activity is an effective strategy for promoting active lifestyles. However, accurately assessing physical activity remains challenging in certain situations. This study evaluates a novel floor-vibration monitoring system to quantify housework-related physical activity.

**Objective:**

This study aims to assess the validity of step-count and physical behavior intensity predictions of a novel floor-vibration monitoring system in comparison with the actual number of steps and indirect calorimetry measurements. The accuracy of the predictions is also compared with that of research-grade devices (ActiGraph GT9X).

**Methods:**

The Ocha-House, located in Tokyo, serves as an independent experimental facility equipped with high-sensitivity accelerometers installed on the floor to monitor vibrations. Dedicated data processing software was developed to analyze floor-vibration signals and calculate 3 quantitative indices: floor-vibration quantity, step count, and moving distance. In total, 10 participants performed 4 different housework-related activities, wearing ActiGraph GT9X monitors on both the waist and wrist for 6 minutes each. Concurrently, floor-vibration data were collected, and the energy expenditure was measured using the Douglas bag method to determine the actual intensity of activities.

**Results:**

Significant correlations (*P*<.001) were found between the quantity of floor vibrations, the estimated step count, the estimated moving distance, and the actual activity intensities. The step-count parameter extracted from the floor-vibration signal emerged as the most robust predictor (*r*^2^=0.82; *P*<.001). Multiple regression models incorporating several floor-vibration–extracted parameters showed a strong association with actual activity intensities (*r*^2^=0.88; *P*<.001). Both the step-count and intensity predictions made by the floor-vibration monitoring system exhibited greater accuracy than those of the ActiGraph monitor.

**Conclusions:**

Floor-vibration monitoring systems seem able to produce valid quantitative assessments of physical activity for selected housework-related activities. In the future, connected smart home systems that integrate this type of technology could be used to perform continuous and accurate evaluations of physical behaviors throughout the day.

## Introduction

There is evidence associating regular physical activity with lower risks for mortality and noncommunicable diseases [1], encouraging researchers, policy makers, and health care companies to develop strategies for the promotion of active lifestyles. The self-monitoring of physical behavior is one approach that has been described as effective for helping people increase their level of physical activity [2-5]. The recent expansion of the activity tracker device market has opened new perspectives for the promotion of active behavior [3,6].

Technology that enables the objective assessment of physical behaviors has evolved considerably over the past decades [7]. Modern activity trackers are generally worn at the waist or the wrist, and while the most recent devices usually feature multiple sensing abilities, the evaluation of physical behaviors mostly results from the treatment of acceleration data acquired by an integrated 3-axis microelectromechanical accelerometer sensor chip [8]. Software tools are able to compute a wide range of parameters related to physical behaviors, such as sedentary time, step count, and estimated energy expenditure [9]. Activity tracker devices can be paired with smartphone apps, transforming smartphone handsets into genuine hubs for the monitoring of physical activity and sedentary behaviors. Although waist- and wrist-worn activity tracker devices have been linked to inaccurate energy expenditure predictions [10-12], the emergence of the 5G and Internet of Things devices opens up room for more accurate and continuous monitoring, where multiple connected devices can collect a wealth of information about people’s physical behaviors throughout the day. In such a connected environment, and while housework-related activities account for a substantial proportion of daily physical activity in some populations [13], smart home systems could provide crucial information to (1) support the continuity of the monitoring of physical activity and sedentary behaviors when people stay at home by allowing them to remove their wearable activity tracker device and (2) improve the accuracy of energy expenditure predictions related to home-based activities. However, although various smart home projects have already included technological features allowing monitoring of the physical behaviors of the occupants, to date, the information has mainly been used as input to smart appliances to adapt to the living environment, assist occupants with disabilities, or optimize domestic energy consumption [14-17]. In these projects, various monitoring technology devices have been considered, including motion sensors, low-resolution video cameras, Kinect systems (Microsoft), and accelerometer-based wearable monitors [16,18-20]. Floors with sensing capabilities have also been developed. For instance, binary pressure detection floor systems have been used to detect the position of occupants [21,22]. Floor geophones and accelerometer sensors have been used to locate footsteps or evaluate room occupancy [23,24]. Unfortunately, none of these projects have prioritized the objective and quantitative assessment of physical behaviors with the ultimate goal of providing lifestyle-oriented feedback to the occupants. Nevertheless, smart home systems capable of monitoring physical activity and sedentary behaviors while providing feedback to the occupants have the potential to encourage individuals to adopt more active and healthier behaviors throughout the day [2-5].

In this study, the floor-vibration measurement system of the experimental Ocha-House was used to collect information about the floor vibrations generated by the occupant and estimate the energy expenditure and step count. A structured experiment consisting of the completion of 4 typical home-based activities was conducted to assess the validity of these estimations with respect to the actual measurements performed by indirect calorimetry (energy expenditure) and direct observation (step count). The estimations of the floor-vibration monitoring system were also compared with those of waist- and wrist-worn research-grade activity tracker devices. It is hypothesized that the floor-vibration monitoring system is capable of correctly predicting energy expenditure.

## Methods

### The Ocha-House Project

**Figure 1:**
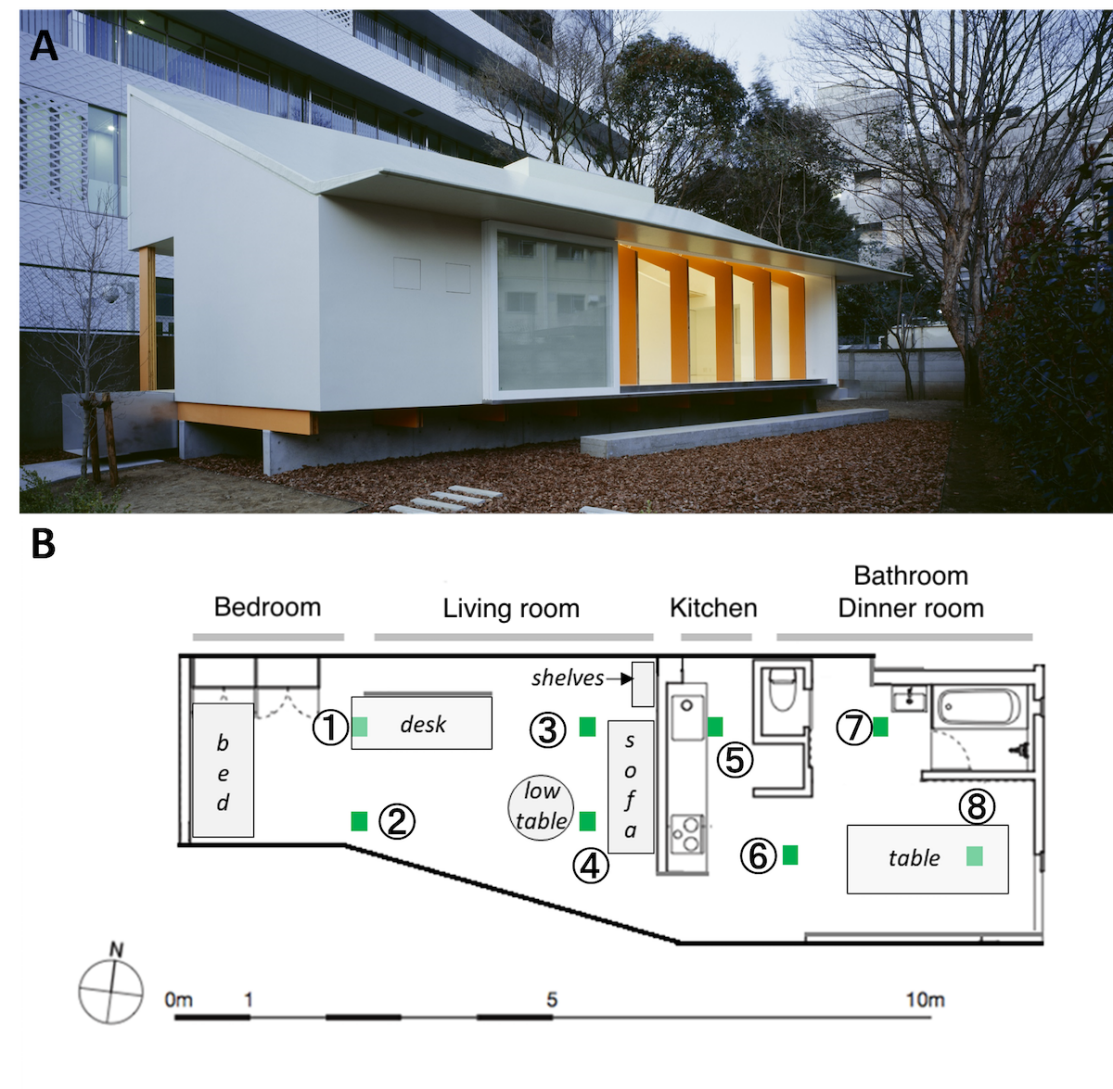
Overview of the Ocha-House and the experimental area. (A) External view of the building (image captured from southwest corner of the yard). (B) Plan of the house and the experimental area, with furniture indicated in light gray, and green squares (labeled 1-8) indicating sensor positions. The house comprises 2 distinct areas separated by a wall. The west side features a fully open space housing a bedroom corner and a living room without any additional partition wall. On the east side, the kitchen and dining room are interconnected through an open space. The toilet and bathroom corners are situated in enclosed spaces on the east side of the building.

The experimental Ocha-House is in the Bunkyo district in the central area of the Tokyo metropolitan region. The project was originally designed as a ubiquitous computing house that allows the mounting of various sensing devices [25]. According to Japanese standards, the Ocha-House corresponds to an extended 1LDK dwelling, that is, a 1-bedroom apartment with a kitchen separated from the living and dining areas. An overview of the building and experimental area is shown in Figure 1.

The experimental Ocha-House is in the Bunkyo district in the central area of the Tokyo metropolitan region (Figure 1A). The total experimental surface area was 42 m^2^ (Figure 1B). A total of 8 high-sensitivity uniaxial accelerometers (shear-type pickup PV-87; Rion Co. Ltd) were installed on the floor to measure the floor vibrations occurring on the experimental surface. The PV-87 sensor characteristics were specified as follows by the manufacturer: charge sensitivity +40 pC to –40 pC/m/s^2^; range of detection 1 to 3000 Hz; dimensions 24 mm (hex) × 30.5 (H) mm; mass 115 g. The optimal number of sensor units and their locations were determined through a series of preliminary experiments. These experiments involved progressively increasing the sensitivity setting of the sensors and the number of units placed on both the west and east sides of the Ocha-House. The operation continued until the coverage was deemed sufficient to detect human motion across all parts of the experimental surface. The data related to these preliminary experiments are not presented here. The sensors were mounted on the floor using double-sided tape, as recommended by the manufacturer, and connected to 4 UV-16 2-channel charge amplifiers (Rion Co Ltd) configured in accordance with the manufacturer’s recommendations. The floor-vibration data acquisition was performed using USB-6008 data acquisition devices (National Instrument Corp), a laptop equipped with MATLAB 2015b, and the necessary data acquisition toolbox (MathWorks Inc). The signal was digitized at a 100-Hz sampling rate with a resolution of 12 bits. The abovementioned system is described hereafter as the *floor-vibration monitoring system*.

### Study Protocol

**Figure 2:**
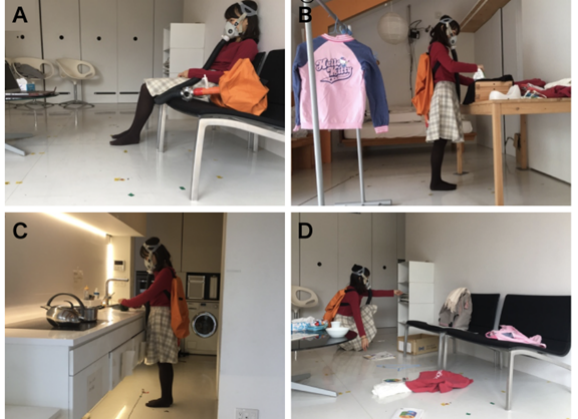
Images of experiments. (A) Sitting and watching videos; (B) ironing, folding, and hanging clothes; (C) cooking, setting the table, and serving food; and (D) cleaning the room.

In total, 10 female participants engaged in 4 activities in the Ocha-House. Participants were recruited from the Ochanomizu University campus, which is a women’s university. They were selected based on the inclusion criterion of being aged ≥18 years, with the exclusion criterion being physical imbalance. Participant characteristics are summarized in Table 1, and the details of the 4 activities performed at the Ocha-House are presented in Figure 2. Before the commencement of the experiment, each participant completed a brief walking trial in the Ocha-House, lasting approximately 1 minute. In total, 2 researchers with expertise in gait analysis visually determined the gait type of each participant, specifically identifying whether they exhibited a heel strike or a lighter mid-strike or forefoot-strike landing. The walking trial revealed that all participants could be categorized into either heel-strike landing or mid-strike or forefoot-strike landing categories, with no other gait types observed. All experiments were conducted without any footwear, including slippers. In total, 9 participants wore socks. Moreover, 1 participant did not wear socks on the day of the experiment and completed the protocol barefoot.

**Table 1:** Participant characteristics (N=10).

		Values
Age (y), mean (SD)		24 (7)
Body weight (kg), mean (SD)		47 (6)
BMI (kg/m^2^), mean (SD)		19 (1)
**Gait type**		
	Heel strike	3
	Mid- or forefoot strike^a^	7

^a^The column “gait type” refers to the number of participants presenting a strong heel strike during the walking gait cycle, as opposed to participants who presented a lighter mid-strike or forefoot-strike landing. The gait type of the participants was visually determined during the walking calibration trial.

In total, 4 activities were selected from the “inactivity quiet/light” or “home activities” categories of the compendium of physical activities [26]. The metabolic equivalent of task (MET) values, indicating the intensity of each activity, were reported in the compendium as follows:

- Sitting and watching videos, hereafter referred to as *sitting* (approximately 1.3 MET, activity code 07020).
- Ironing, folding, and hanging clothes, hereafter referred to as *ironing* (1.8-2 METs, activity codes 05070 and 05090).
- Cooking, setting the table, and serving food, hereafter referred to as *cooking* (approximately 2.5 METs, activity codes 05051 and 05052).
- Cleaning the room, hereafter referred to as *cleaning* (approximately 3.3 METs, activity code 05030).

Each activity lasted 6 minutes. To balance the contribution of the 3 tasks within “cooking, setting the table, and serving food” and considering the volume of the Douglas bag used for energy expenditure measurement (see the section Indirect Calorimetry), participants were orally given time information. This ensured that they spent approximately 40 seconds on each task every 2 minutes. The floor-vibration monitoring system recorded floor vibrations, estimate the number of steps, and compute quantitative parameters as described in the Floor Vibration Signal Treatment and Data Feature Extraction section. The participants wore 2 ActiGraph GT9X monitors (ActiGraph LLC) at the waist and wrist. The 10-second epoch activity count and the step-count prediction were recorded for each activity. Finally, Douglas bags were used during the last 2 minutes of each activity to collect the air expired by the participants, perform indirect calorimetry measurements, and obtain the actual energy expenditure. Throughout the experiments, the researchers stood quietly on an insulated part of the floor outside the experimental area to avoid producing confounding vibrations. The experiments were video recorded (data not shown).

### Ethical Considerations

The experimental protocol was approved by the Ochanomizu University Research Ethics Committee (2018-18). All the participants provided written informed consent and they did not receive any compensation. The individual shown in Figure 2 provided informed consent for the publication of their image.

### Floor-Vibration Signal Treatment and Data Feature Extraction

The 8-sensor floor-vibration data of each 6-minute activity corresponded to 8 time series of 36,000 samples. Raw data were expressed in volt. For each series, the floor-vibration signal was rectified and smoothed using a Butterworth filter. The vector norms of the 8 sensors were then computed. A location-based calibration coefficient was applied to the vector norm for each data sample to uniformize the vibration magnitudes throughout the experimental surface (Multimedia Appendix 1). The 3 following data features were extracted:

- Floor count—for each participant and each activity, 36,000 sample values (6-min ×100 Hz sampling rate) of the uniformized vector norm time series were summed to obtain the *floor count* parameter.
- Step count—data from the uniformized vector norm time series were cut in windows of 1 second with an overlap of 50%. For each window, the number of steps was computed using a standard peak detection algorithm configured to detect vibration peaks with a minimum prominence of 1.05 SD and a minimum interval of 250 ms [27]. The *step count* parameter was computed for each activity of each participant by summing the number of unique peaks throughout the activity (6 min). The step count parameter was used as both a data feature, allowing the prediction of energy expenditure and a physical activity parameter to be compared with the direct observations and the outcomes of the waist- and wrist-worn ActiGraph devices.
- Moving distance—the uniformized vector norm time series was cut into windows of 1 second and averaged for each window. The window average was compared with a criterion value calculated for each individual from the data collected during the walking trial to determine whether the participant was moving. Subsequently, the filtered data of the 8-sensor time series corresponding to the windows where the participant was moving were used to compute their location in the house. The distances between all locations taken sequentially were summed for each activity to obtain the *moving distance* parameter (6 min). This parameter was tested and validated against the moving distance estimated from the observation of the video records (Figure S1 in Multimedia Appendix 2).

**Figure 3:**
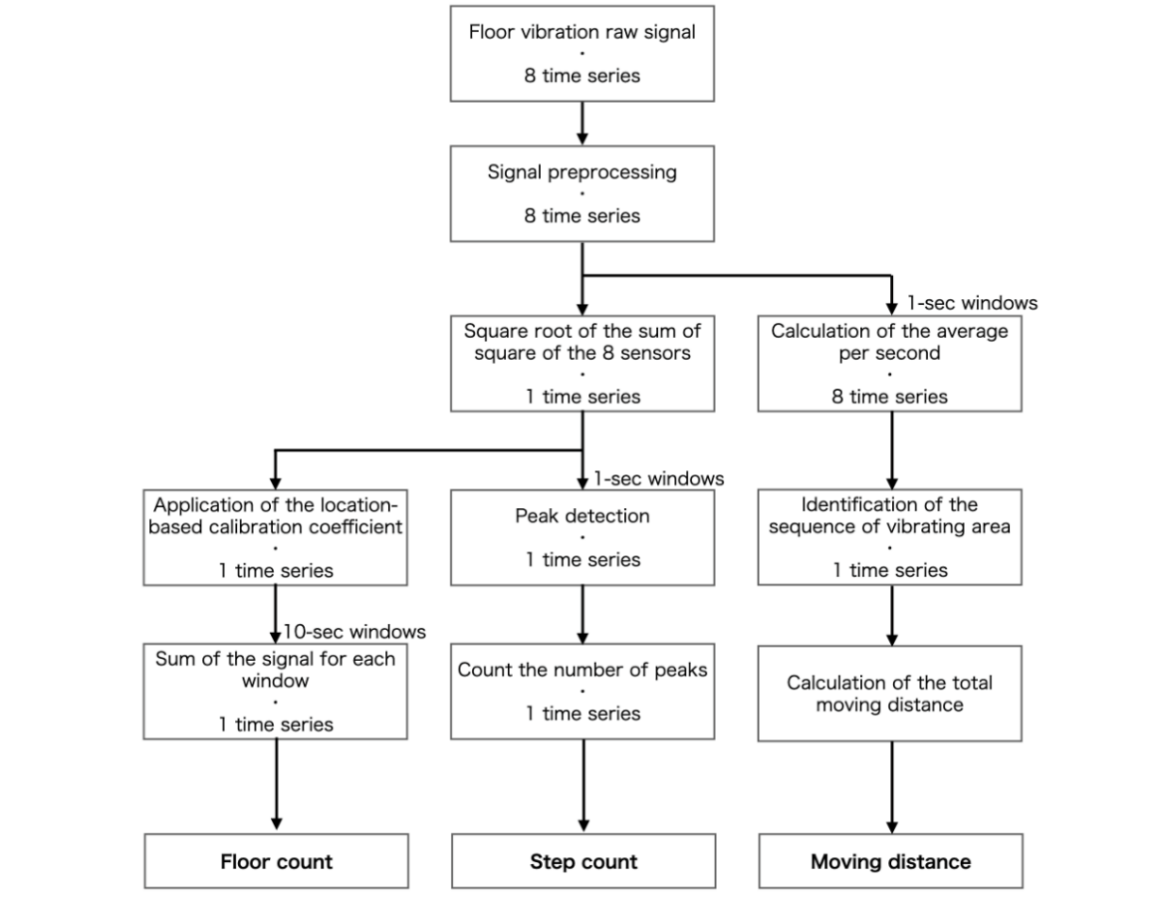
Floor-vibration signal flow processing chart. From 8 time series of raw signals to the extraction of 3 floor-vibration–based data features.

The signal treatment and computation of the 3 previously described parameters were performed using the toolboxes included in the SciPy library [28]. An overview of the entire data processing process is shown in Figure 3.

### Actigraphy

The participants were equipped with 2 ActiGraph GT9X monitors worn on the waist and the wrist. The waist-worn device was positioned near the right hip of the participant, on the belt, or on the upper edge of the skirt or the bottom of the trousers. An elastic belt provided by the manufacturer was used when the clothes worn by the participant did not allow the tight mounting of the monitor. The wrist-worn GT9X device was mounted tightly on the nondominant hand always in the same direction. The 2 monitors were mounted by the same experimenter for all participants. The ActiGraph monitor data were collected in 1-second epochs. The 10-second epoch *activity* count data were extracted from the wrist-worn as well as waist-worn devices, and the “Crouter adult (2010)” equation was used to compute energy expenditure predictions (ActiLife 6; ActiGraph LLC) [29]. This algorithm uses a refined 2-regression model to distinguish between walking and lifestyle activities. In this study, the activity intensities were expressed in MET (ie, energy expenditure/participant weight/6 min). The number of steps estimated by the waist- and wrist-worn monitors was also recorded.

### Indirect Calorimetry

The actual energy expenditure was measured using the Douglas bag method during the last 2 minutes of each activity. The air composition of the bags was analyzed using a mass spectrometry gas analyzer (ARCO-1000; Arco System) calibrated on each experimental day in accordance with the manufacturer’s instructions. The gas volume was determined using a gas meter (DC-5; Shinagawa). The energy expenditure was estimated from oxygen consumption (VO_2_) and carbon dioxide production (VCO_2_) using the Weir formula (ie, 3.9VO_2_+1.1VCO_2_). In addition, the resting metabolic rate of each participant was evaluated before the experiment. The participant lay for 15 minutes on the bed of the Ocha-House, and the expired air was collected during the last 2 minutes. The actual MET value for each activity was calculated as the activity energy expenditure divided by the resting metabolic rate.

### Video Recording

The experimental sessions were video recorded using an Arrows M03 smartphone (Fujitsu Ltd) or an iPad Mini 3 (Apple Inc). In total, 2 independent investigators inspected the videos and counted the number of actual steps for the 4 activities of each participant. In this study, a “step” is defined as the shift of the body weight support from one leg to the other, which includes a single-leg support phase and occurs at least partially on the anterior-posterior axis.

### Statistical Analysis

The 4 activities were compared for the *floor count*, *step count*, *moving distance* parameters using an ANOVA or the Kruskal-Wallis test, and multiple comparison procedures (Tukey or Nemenyi tests) were performed to locate differences. The same analysis was conducted for parameters extracted from the waist- and wrist-worn GT9X monitors and for the indirect calorimetry measurements. The relationships between the actual intensities measured by indirect calorimetry and *floor count*, *step count*, and *moving distance* were investigated using single linear regression tests. Multiple regression models were used to explore the relationship between different combinations of descriptors, including *floor count*, *step count*, and *moving distance*, and the actual intensities measured using indirect calorimetry. This analysis was conducted hierarchically. First, the regressions were only performed on the floor-vibration–extracted parameters. Second, the participant characteristics (ie, body weight and gait type) were included in the model descriptors. Additional hierarchical models are presented in Multimedia Appendix 2. The model with the highest *R*^2^ value best explained the variation in the data and was selected for subsequent testing. Finally, mixed model ANOVA and post hoc pairwise operations were used to compare the performance of the best models built on data obtained with the floor-vibration–based monitoring system, the waist- and wrist-worn GT9X monitors, against the actual intensity and the actual number of steps.

The underlying assumptions for each test were evaluated before conducting the analyses. Data are presented as mean (SD). The statistical analysis was performed using the following Python libraries: StatsModels (0.13.2), Pingouin (0.5.3), and Scikit-Posthocs (0.7.0) [30,31]. Data used for the statistical analysis are shared in the Multimedia Appendix 3.

## Results

### Actual Intensities and Number of Steps

**Figure 4:**
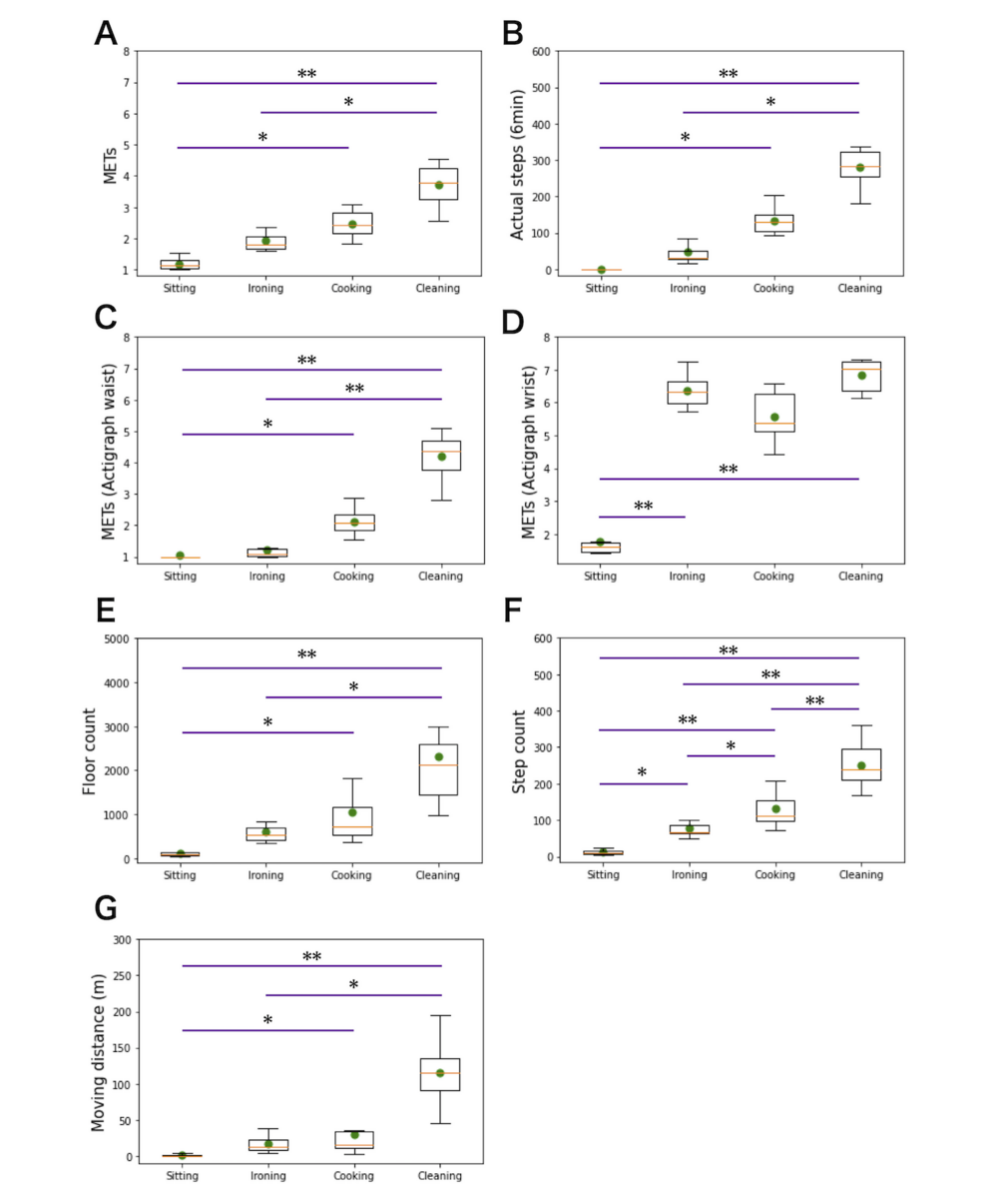
Comparison between the 4 experimental home activities. (A) actual intensities (indirect calorimetry evaluation); (B) actual number of steps (video observation); (C) activity intensity predicted by the waist-worn ActiGraph GT9X device; (D) activity intensity predicted by the wrist-worn ActiGraph GT9X device; (E) floor-vibration–based computed floor-count; (F) floor-vibration–based computed step-count; (G) floor-vibration–based computed moving-distance. The intensity predictions of the GT9X monitors were computed using the “Crouter adult (2010)” equation [29]. Yellow line: median. Green point: average. Outliers are not depicted. MET: metabolic equivalent of task. *P<.05, **P<.001.

Activity intensities calculated from indirect calorimetry measurements were as follows: 1.2 (SD 0.2) MET for the sitting behavior, 1.9 (SD 0.4) MET for the ironing activity, 2.5 (SD 0.4) MET for the cooking activity, and 3.7 (SD 0.6) MET for the cleaning activity (Figure 4A). Pairwise comparisons indicated significant differences in intensity between *sitting* and *cooking*, between *sitting* and *cleaning,* and between *ironing* and *cleaning* (*P*=.004, *P*<.001, and *P*=.01, respectively). The actual number of steps was 0 (SD 0) for *sitting*, 48 (SD 36) for *ironing*, 133 (SD 35) for *cooking*, and 281 (SD 48) for *cleaning* (Figure 4B). Pairwise comparisons indicated significant differences in steps between *sitting* and *cooking*, between *sitting* and *cleaning*, and between *ironing* and *cleaning* (*P*=.003, *P*<.001, and *P*=.003, respectively).

### Floor Count, Step Count, and Moving Distance Parameters Computed From Floor Vibrations

The *floor count* parameter (arbitrary units) was as follows for each activity: 113 (SD 58) for *sitting*, 610 (SD 277) for *ironing*, 1046 (SD 748) for *cooking*, and 2323 (SD 1255) for *cleaning* the room (Figure 4E). Pairwise comparisons indicated significant differences in intensity between *sitting* and *cooking*, between *sitting* and *cleaning*, and between *ironing* and *cleaning* (*P*=.006, *P*<.001, and *P*=.04, respectively). The estimated number of steps was as follows: 12 (SD 7.3) for *sitting*, 78 (SD 27) for *ironing*, 133 (SD 53) for *cooking*, and 251 (SD 59) for *cleaning* (Figure 4F). Pairwise comparisons indicated that the respective intensities of the 4 activities were all significantly different (between sitting and ironing, *P*=.01; between ironing and cooking, *P*=.04; and others, *P*<.001). Finally, the *moving distance* parameter scored as follows: 1.3 (SD 1.8) m for *sitting*, 17 (SD 11) for *ironing*, 30 (SD 29) for *cooking*, and 115 (SD 39) for *cleaning* (Figure 4G). Pairwise comparisons indicated significant differences in intensity between *sitting* and *cooking*, between *sitting* and *cleaning*, and between *ironing* and *cleaning* (*P*=.03, *P*<.001, and *P*=.03, respectively). Furthermore, a significant correlation was found between the *moving distance* outcomes computed from the floor-vibration signal and the moving distances estimated from the video records (Figure S1 in Multimedia Appendix 2).

### Actigraphy

The waist-worn activity tracker estimated the activity intensities as follows: 1.1 (SD 0.2) MET for *sitting*, 1.2 (SD 0.3) MET for *ironing*, 2.1 (SD 0.4) MET for *cooking*, and 4.2 (SD 0.7) MET for *cleaning* (Figure 4C). Pairwise comparison analyses indicated significant differences between *sitting* and *cooking*, between *sitting* and *cleaning*, and between *ironing* and *cleaning* (*P*=.01, *P*<.001, and *P*<.001, respectively). The estimated number of steps were as follows: 0.9 (SD 2.4) for *sitting*, 8 (SD 12) for *ironing*, 55 (SD 27) for *cooking*, and 165 (SD 51) for *cleaning* (Multimedia Appendix 2). Pairwise comparison analyses indicated significant differences between *sitting* and *cooking*, between *sitting* and *cleaning*, and between *ironing* and *cleaning* (*P*=.007, *P*<.001, and *P*<.001, respectively).

The wrist-worn activity tracker estimated the activity intensities as follows: 1.8 (SD 0.4) MET for *sitting*, 6.4 (SD 0.5) MET for *ironing*, 5.6 (SD 0.7) MET for *cooking*, and 6.8 (SD 0.4) MET for *cleaning* (Figure 4D). Pairwise comparisons indicated significant differences between *sitting* and *ironing* and between *sitting* and *cleaning* (*P*=.002 and *P*<.001, respectively). The estimated number of steps was as follows: 11 (SD 5.7) for *sitting*, 177 (SD 39) for *ironing*, 131 (SD 37) for *cooking*, and 197 (SD 35) for *cleaning* (Multimedia Appendix 2). Pairwise comparison analyses indicated significant differences between *sitting* and *ironing* and between *sitting* and *cleaning* (*P*<.001 for both).

The results for the activity count parameters of the waist- and wrist-worn devices are shown in Multimedia Appendix 2.

### Relationship Between the Floor-Vibration–Based Outcomes and the Actual Activity Intensities

As presented in Table 2, *floor count*, *step count*, and *moving distance* were significantly associated with the intensity of physical behavior (*r*^2^=.56, *r*^2^=.82, *r*^2^=.66, respectively; *P*<.001). Combining the 3 parameters resulted in an *r*^2^ value of 0.84. Combining *floor count*, *step count*, and *moving distance* with participant personal characteristics, such as body weight and gait type, allowed predicting the intensity of physical behaviors with an accuracy of 88% (Table 1). The results of the additional hierarchical models are presented in Multimedia Appendix 2.

**Table 2:** Relationship between floor-vibration–based parameters and actual activity intensities evaluated by indirect calorimetry.

Models^a^ and predictor variables				Standardized partial regression coefficient		*P* value		*r* ^2^
**Single regressions**								
	1	Floor count	0.745		<.001		0.56	
	2	Step count	0.904		<.001		0.82	
	3	Moving distance	0.815		<.001		0.66	
**Multiple regressions**								
	**4**							
		Step count	0.971		<.001		0.85	
		Floor count	–0.43		.01		0.85	
		Moving distance	0.361		.02		0.85	
	**5**							
		Step count	0.916		<.001		0.88	
		Moving distance	0.259		.09		0.88	
		Floor count	−0.224		.20		0.88	
		Body weight	−0.08		.21		0.88	
		Gait type	−0.155		.03		0.88	

^a^Regression models combining 2 vibration parameters with and without participant characteristic parameters are shown in Multimedia Appendix 2. “Gait type” is binary data (mid- or forefoot strike vs heel-strike foot landing).

### Step Count and Activity Intensity Predictions

The mixed model ANOVA showed a significant effect of the measurement method and a significant interaction with the activity for both the predictions of the number of steps and activity intensities (*P*<.001).

**Figure 5:**
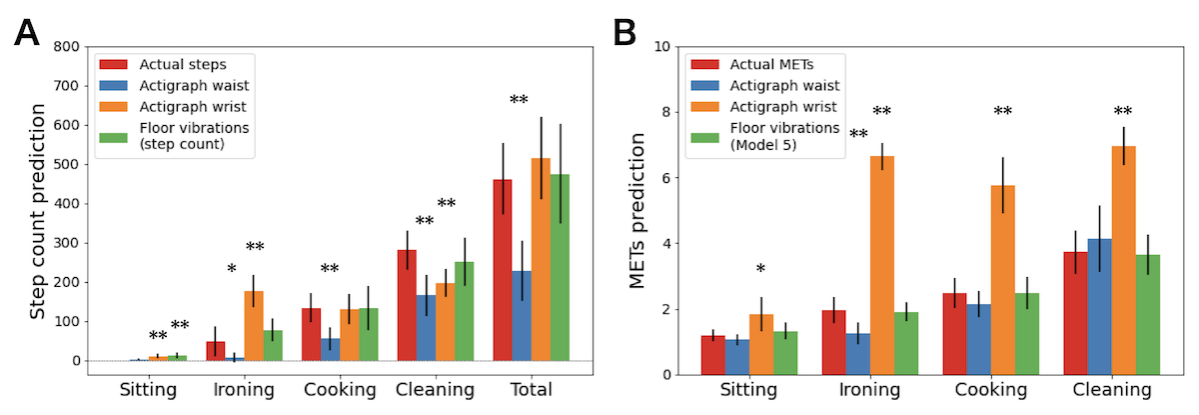
Comparison of prediction methods for each activity. (A) number of steps; (B) activity intensity. Energy expenditure measurements were collected for 2 minutes over the 6 minutes of each activity. Therefore, comparisons of measurement methods for the total energy expenditure are not presented in this figure. In panel B, the green bar shows the prediction of the model with the highest coefficient of determination (model 5; Table 2). The marks indicate a significant difference against the actual number of steps. MET: metabolic equivalent of task. *P<.05, **P<.001.

Significant underestimations in the number of steps were noted for the waist-worn ActiGraph device predictions when all activities were considered together (Figure 5A, “total”). Regarding the prediction of activity intensities (Figure 5B), the pairwise comparison analyses revealed statistically significant overestimations for the wrist-worn ActiGraph device across all activities, The wrist-worn device and the floor-vibration system did not exhibit any difference with the actual number of steps. When activities are considered separately, pairwise comparisons indicate that the wrist-worn ActiGraph device underestimated the number of steps completed during *cleaning* but overestimated it for *sitting* and *ironing* activities. The floor-vibration–based predictions overestimated the number of steps required for *sitting*.

Regarding the prediction of activity intensities, the pairwise comparison analyses revealed statistically significant overestimations for the wrist-worn ActiGraph device across all activities. The floor-vibration system did not show any differences with the actual intensities evaluated by indirect calorimetry. Finally, the waist-worn ActiGraph device showed a slight but significant underestimation for the estimations of the *ironing* activity intensities. The results are shown in Figure 5.

## Discussion

### Principal Findings

This study presented a novel quantitative method that uses the monitoring of floor vibrations for the evaluation of physical behaviors at home. The *floor count*, *step count*, and *moving distance* parameters were computed from the floor-vibration signal. Statistical models combining these 3 parameters showed significant correlations with the actual energy expenditure measured in 10 participants in a structured experiment that included 4 common home-based activities. In addition, the *step count* parameter did not show any significant difference with the number of steps completed by the participants during the experiment. The predictions of the floor-vibration monitoring system for both the activity intensity and number of steps were equal to or more accurate than those obtained by the Actigraphy method using the refined 2-regression model.

### Floor Vibration for the Quantification of Physical Behaviors

The actual activity intensities measured by the indirect calorimetry for the 4 activities increased in accordance with the intensities presented in the compendium of physical activities [26], that is, 1.2 versus 1.3 MET; 1.9 versus 1.8 to 2.0 MET; 2.5 versus 2.5 MET; and 3.7 versus 3.3 MET for *sitting* (and watching video), *ironing* (and folding clothes), *cooking* (and setting the table), and *cleaning* the room, respectively (Figure 4A). The *floor count*, *step count*, and *moving distance* parameters also increased gradually for the 4 activities, indicating the feasibility of evaluating the intensity of physical behaviors in the home environment using the information provided by floor vibrations (Figures 4E-4G). Among them, *step count* was the only parameter that showed significant differences between all 4 activities. The single regression analyses also indicated a strong association between the *step count* and the actual activity intensities (*r*^2^=0.82; *P*<.001), whereas *floor count* and *moving distance* showed a weaker but still significant association with the actual activity intensities (*r*^2^=0.56 and 0.66, respectively; *P*<.001 for both). These pieces of information taken together may suggest that the estimated number of steps extracted from the floor-vibration signal could be used to make a reliable quantitative estimation of physical behavior in home settings. All the 3 parameters were designed to describe the inhabitant’s motion. Although the *step count* and *floor count* parameters can capture the physical dimension of the movement, the *moving distance* parameter adds a spatial dimension to the evaluation. The better performance of *step count* alone compared with *moving distance* alone may be due to the location approximation inherent to the limited number of sensors used to cover the entire house surface (Multimedia Appendix 2). A step is detected and not affected by any approximation. On the other hand, *floor count* may also be susceptible to inaccuracies, potentially due to variations in floor-vibration wave attenuation. Factors influencing this attenuation include the proximity of furniture, its weight and contact surface with the floor, proximity of support beams and walls, and the irregular geometry of the Ocha-House floor area.

Despite the possible weaknesses of the *floor count* and *moving distance* parameters, multiple linear regression models combining *step count, floor count*, and *moving distance* still exhibited stronger associations (*r*^2^≥0.85; Table 1; Table S1 in Multimedia Appendix 2). As presented in Table 2, the inclusion of body weight and gait type parameters as descriptors greatly lowered the contribution of *floor count* to the model. Indeed, although *floor count* does not correlate with either body weight or gait type (Table S2 in Multimedia Appendix 2), it is still the only parameter extracted from floor vibrations that can quantitatively capture the forces applied on the floor. Given that the actual body weight can be easily inputted into any smart home system, the question of the relevance of extracting and using the *floor count* parameter to make accurate predictions of energy expenditure remains open. Additional studies, including a population with more heterogeneous anthropometric characteristics, may be needed to address this question further.

Finally, the ANOVA revealed that the predictions made by the floor-vibration monitoring system also showed less deviation than those of the 2 research-grade ActiGraph GT9X monitors for both the number of steps and activity intensity end points (Figure 5). The underestimation of the number of steps noted for the waist-worn ActiGraph device may be related to walking gait characteristics when movements are performed in closed and narrow spaces. Such environments may not allow sufficient acceleration to meet the necessary signal processing threshold criteria required to count a step, as suggested elsewhere [32]. In contrast, the overestimated number of steps observed for the wrist-worn device may be the result of confounding upper limb movements performed in a frequency range similar to that of walking gait, which may occur in the course of completing housework-related activities.

Regardless of the performance of the ActiGraph GT9X monitors and although no external validation experiment has been conducted, taken together, these observations emphasize the good performance of the floor monitoring system for the quantitative evaluation of physical behaviors performed in home settings.

### Perspectives

Although the market for wearable activity trackers is still in its growing phase [6], waist- and wrist-worn physical activity monitors, including research- or consumer-grade devices, have been associated with inaccurate predictions of daily physical activity [10-12]. During the past decade, the computation of accurate predictions for housework-related activities using traditional accelerometer-based activity tracker devices has been the object of specific software development [33,34]. However, this study still showed statistical differences between the predictions made by the ActiGraph GT9X monitors and the actual values for both number of steps and activity intensities. These observations emphasize the necessity of developing new methods that can accurately evaluate physical behaviors at home to improve the computation of daily physical activity metrics. When considering long-term use, it is crucial to distinguish between consumer- and research-grade devices. This study used 2 ActiGraph GT9X monitors, recognized as research-grade devices, in the context of a short semistructured experiment. However, consumer-grade physical activity tracking devices used in everyday life are subject to additional extrinsic limitations that can impede their ability to provide continuous monitoring. For instance, the common practice of removing watches and other wearables at home can have significant impacts on the evaluation of physical behaviors in home settings.

Considering the current limitations of wearable activity tracker devices, smart home systems, such as floor-vibration monitoring technologies similar to those used in this study, present a suitable opportunity to enhance the self-monitoring of daily physical activity. Such systems offer a novel approach to improve the accuracy of estimating energy expenditure and the number of steps performed at home, especially when considering their integration with a 5G network composed of interconnected devices dedicated to the evaluation of daily physical activity. By ensuring accurate and continuous measurements when individuals are at home, smart home systems could help maintain people’s interest in self-monitoring, making them a pivotal factor in promoting and sustaining active and healthy lifestyles. However, the potential widespread adoption of such systems should not only be considered from a technological perspective but should also acknowledge the role of sociocultural factors in shaping user acceptance and usability.

### Limitations and Strengths

The main limitation of this study is that the proposed method only assesses the physical behaviors of 1 inhabitant at a time. Quantifying the physical behaviors of multiple individuals would require additional signal processing tools to link vibration events with the individuals generating them. Although each individual may exhibit a unique gait signature reflected in the floor-vibration signal, extracting such information is beyond the scope of this study. This limitation could also be addressed by analyzing the sequence of interactions with smart and connected home furniture devices, similar to what has already been described elsewhere [35]. Furthermore, it is important to note that the experimental Ocha-House used in this study was originally designed for a single inhabitant, aligning with the living environment of millions of Japanese people. The structured nature of the experimental protocol may be cited as a second limitation of the study, which restricts the generalization of the observations to what may happen under free-living conditions. To further evaluate the feasibility of using the floor-vibration–based monitoring method, semistructured experiments using a portable breath-by-breath gas exchange analyzer could be conducted to assess energy expenditure during longer periods of activity. A third limitation is that the external validity of the floor-vibration parameter–based activity intensity prediction models (Table 2) was not tested, thus mitigating the interpretations of the comparison test performed against the actigraphy method. The cost of the system is also considered to be a limitation. In this study, expensive, high-sensitivity shear-type accelerometer sensors were used. Further studies are necessary to explore the feasibility of using cheaper accelerometer sensors similar to those commonly used in wearable devices. The results of the multiple regression analyses (Table 2; Multimedia Appendix 2) indicated that the floor-vibration–based *step count* and *moving distance* parameters contributed more to the activity intensity prediction models. These 2 parameters may not require the computation of a high-resolution signal. Fourth, the quasi-absence of responses in the *floor count*, *step count*, and *moving distance* parameters during *sitting and watching videos* may suggest that the floor-vibration monitoring system may also be capable of evaluating sitting behaviors (Figures 4E-Figures 4G). However, owing to the structured nature of the protocol, which involves short observation windows, further interpretations regarding the accuracy of the system in predicting energy expenditure for home-based sitting activities cannot be made. Given the importance, complexity, and intricacies of sedentary behaviors that can occur at home, specific studies should be designed to understand how floor-vibration monitoring systems may contribute to the objective assessment of home-based sedentary behaviors. Finally, the participants in this study were all women and exhibited relatively homogeneous characteristics in terms of age and weight. The BMI scores indicated a limited variability in physical fitness. These observations constrain the generalizability of our results to a more diverse population. Given that age and physical fitness are recognized determinants of energy expenditure, future investigations should aim to recruit a more diverse sample of participants and consider a broader spectrum of personal characteristics in the development of energy expenditure prediction models.

This study has several strengths and originalities. First, the results of the present experiment are in line with those of previous studies, which described a good relationship between the force exerted by an individual on the floor of a small squared 6.25 m^2^ metabolic chamber equipped with a force transducer and the actual energy expenditure [36-38]. They allow extending the previous observation to a different sensing technology, larger non–squared living surfaces, and a wider range of activities that are usually performed at home. Another strength of this study is that it is the first to compare the outcomes of a smart home system with those of research-grade activity trackers.

### Conclusions

This study presents a novel floor-vibration monitoring system that can be used in smart home settings to quantify physical activity at home. The hardware included high-sensitivity accelerometers. In this case, 8 sensors were required to cover a surface area of 42 m^2^. The software includes a simple data processing workflow for the computation of the *floor count* parameter, which is a quantitative index of the floor vibrations, and the *step count* and *moving distance* parameters. Regression models combining the information of these 3 parameters showed a strong association with the actual intensities measured using indirect calorimetry for the 4 tested home-based activities. A significant association was also observed between the *step count* parameter computed using the floor-vibration signal and the actual number of steps. Further studies, conducted under real-life conditions or using semistructured experimental protocols, are necessary to extend the results of this study and validate the monitoring of floor vibrations as a surrogate method for evaluating physical behaviors at home. Considering the current evolution of 5G technologies and IoT devices, smart home systems are expected to contribute to a more continuous evaluation of daily physical activity.

## Appendix

Multimedia Appendix 1 Supplementary descriptions and results of the calibration and walking trials.

Multimedia Appendix 2 Supplementary analyses and results.

Multimedia Appendix 3 Data used for the statistical analysis.

## Abbreviations

MET: metabolic equivalent of task
