# Supplementary material for: Objective Assessment of Physical Activity at Home Using a Novel Floor-Vibration Monitoring System: Validation and Comparison With Wearable Activity Trackers and Indirect Calorimetry Measurements
Source: JMIR Form Res. 2024 Apr 25;8:e51874. doi: 10.2196/51874 (PMC11082727; doi:10.2196/51874)
Supplement: Multimedia Appendix 1 [file formative_v8i1e51874_app1.docx]

**Multimedia Appendix 1**

**Calibration trial**

Preliminary experiments realized in the *Ocha-House* prior to the study presented in the main article had shown variations in the floor-vibration magnitude depending on the participant location in the house. Therefore, a convenient calibration procedure aiming at equalizing the vibration signal magnitude over the whole house surface has been developed. The following sections describe the development of the calibration procedure and gives some selected examples of calibrated data.

*1/ Measurement of the foot landing impact force on floor during walking gait.*

Measurements realized in 5 participants using a floor reaction force meter (Kistler Instrumente AG, Winterthur, Switzerland) showed that the impact forces resulting from foot landing range from 200 to 450 N during walking and can go up to 900 N when jumping. During this walking experiment, all gait impact forces >320 N had been obtained from one outlier participant weighting > 80 kg (average weight of participants engaged in the main experiment: 47±5.8 kg). Four-hundred-fifty newton was therefore considered an upper limit, latter for the calibration procedure. Sample results corresponding to the walking and jumping impact force data of one selected participant are shown in Figure 1.


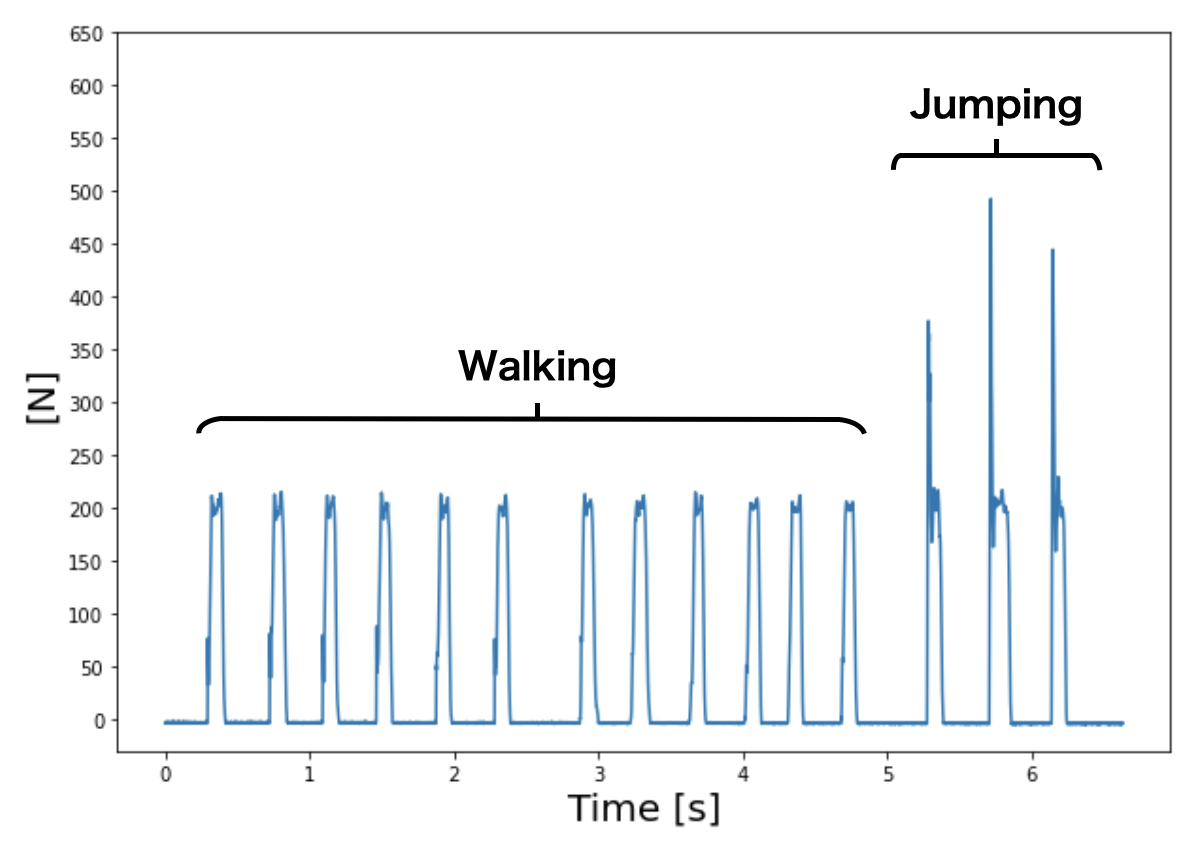
**Figure 1. Signal recorded during gait and jumping on the floor reaction force platform in one selected participant.** Illustration taken from [1].

*2/ Reproduction of the same impact force using a soft rubber ball.*

A convenient protocol allowing dropping a soft rubber ball (Medicine Ball 5 kg; BODYMAKER, Osaka, Japan) from 5, 14, 25, 37 and 48 cm was designed according to what was described in [2]. These five heights allowed reproducing impact forces ranging from 160 to 450 N. Figure 2 describes the relationship between the dropping height and the reaction force measured by the floor reaction force meter.

**
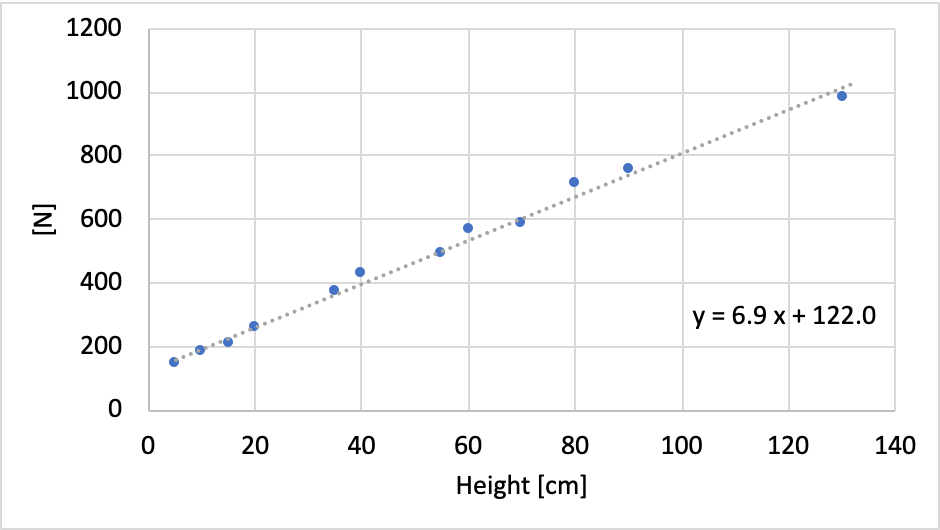
**

**Figure 2. Relationship between the rubber ball dropping height and the impact force.** Illustration taken from [1].

*3/ The Ocha-House floor calibration experiment.*

The calibration experiment consisted in dropping the same soft rubber ball at regular intervals of 1 meter and from the five selected heights, according to the method described elsewhere [2]. The floor vibration data were collected using the 8-sensor system described in the article. Signal rectification and smoothing were performed similarly to what was indicated in the article. Figure 3 shows the thirty-seven 1-m interval calibration points.

**
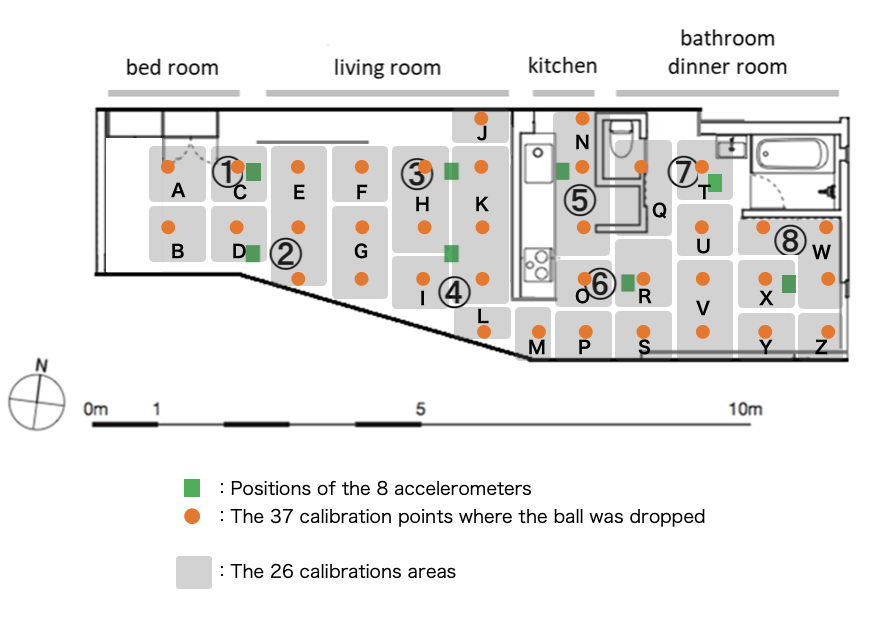
**

**Figure 3. Overview of the Ocha-House experimental surface and the locations of the twenty-six calibration areas relatively to the thirty-seven calibration points.** The 8 sensors are identified with number from 1-8. The 26 calibration areas are identified with letter from A-Z.

At each calibration point and for each dropping height, three measurements were performed. For each measurement and for each sensor, the peak value corresponding to the impact of the rubber soft ball on the *Ocha-House* floor was identified. The sensors were ranked according to their average peak magnitude values and additional signal features were extracted allowing identifying areas that had a similar response. The thirty-seven calibration points were finally categorized in the 26 calibrations areas showed in Figure 3. The *Ocha-house* floor reaction obtained from the 8 accelerometers was considered similar within each of these 26 calibration areas.

For each of the twenty-six areas, the relationship between the ball impact force and the vector norm computed from the data of the 8 sensors was modelled using a polynomial curve fitting. Figure 4, shows two examples of polynomial fittings between impact forces and floor vibration responses, in areas E and U, respectively.


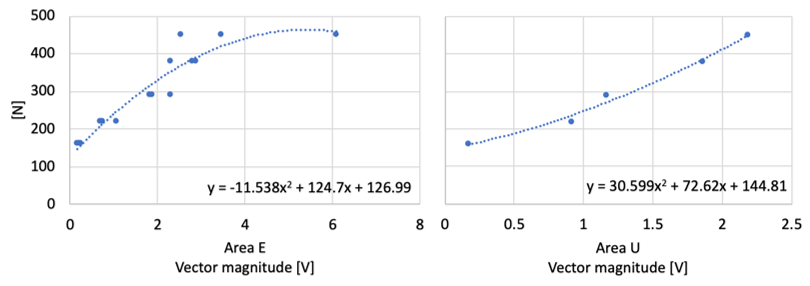


**Figure 4. Relationship between the impact force and the floor vibration observed on the vector norm times series for two selected areas.** (Left panel) calibration area E. (Right panel) calibration area U.

*4/ calibration of the experimental data*

In the method section, cf. “floor vibration signal treatment and data feature extraction”, the article states that “a location-based calibration equation was applied to the vector norm at each data sample in order to uniformize vibration magnitudes throughout the experimental surface”. The present section describes the application of the location-based calibration coefficients. As described in the previous section there are twenty-six coefficients corresponding to the twenty-six areas presented above. The raw floor vibration signal was filtered and smoothed. The average magnitudes of the eight sensors, respectively, were used to rank the sensors and identified the calibration area. The vector magnitude of the eight sensors were calculated and the calibration equation corresponding to the area was used to convert the value to an estimated force within the 0-450 N range. Finally, the converted value was divided by 450 in order to obtained the calibrated floor vibration data, also called “uniformized vector norm” in the article. Therefore, the *uniformized vector norm* data are expressed on a scale going from 0 to 1 consisting in arbitrary unit. The relationship between the first calibration and the zero point was considered linear to “uniformized” data that felt below the 160-450 N.

Figure 5 showed one selected example of the vector norm data collected in participant 2 during the walking trial before and after the uniformization process. Considering the data of the 10 participants, the application of the calibration coefficients allowed reducing the variance of the signal by 45%.

**
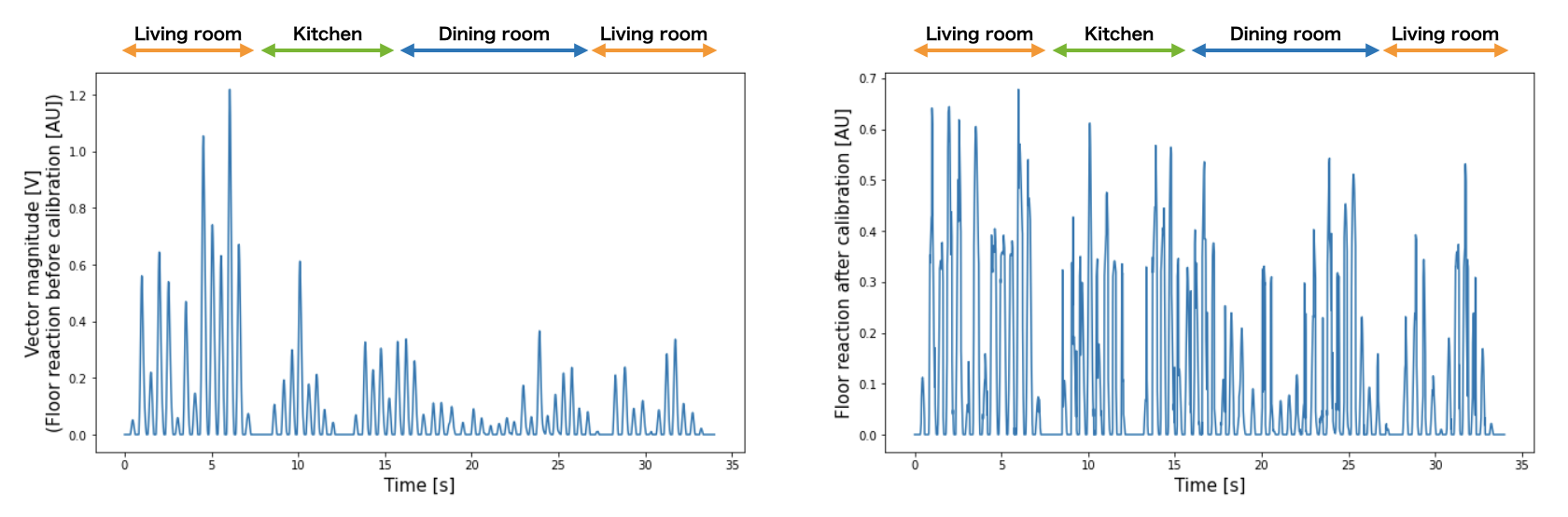
**

**Figure 5. One selected example of the vector norm data before and after the uniformization process applied on the walking trial data, participant 2.** (A) Vector magnitude before the application of the calibration coefficients. Step performed on the North side of the living room generate a higher signal compared to other areas, leading to potential overestimation of the floor count parameter when the inhabitant moves in this area of the house. (B) Uniformized vector magnitude after the application of the calibration coefficients (arbitrary unit). The signal is uniformized throughout the house surface, lowering the influence of the area on the floor-count parameter. The 35-second walking trial data are presented here for the sake of simplicity. However, the procedure has been applied to all experimental data.

**Walking trial**

Before each experiment, the participant performed a “walking trial” consisting in walking in the *Ocha-House* following the path showed in Figure 6. The path was chosen to go near all the 8 sensors. The trial was video recorded and the floor vibration signal was collected for each sensor.


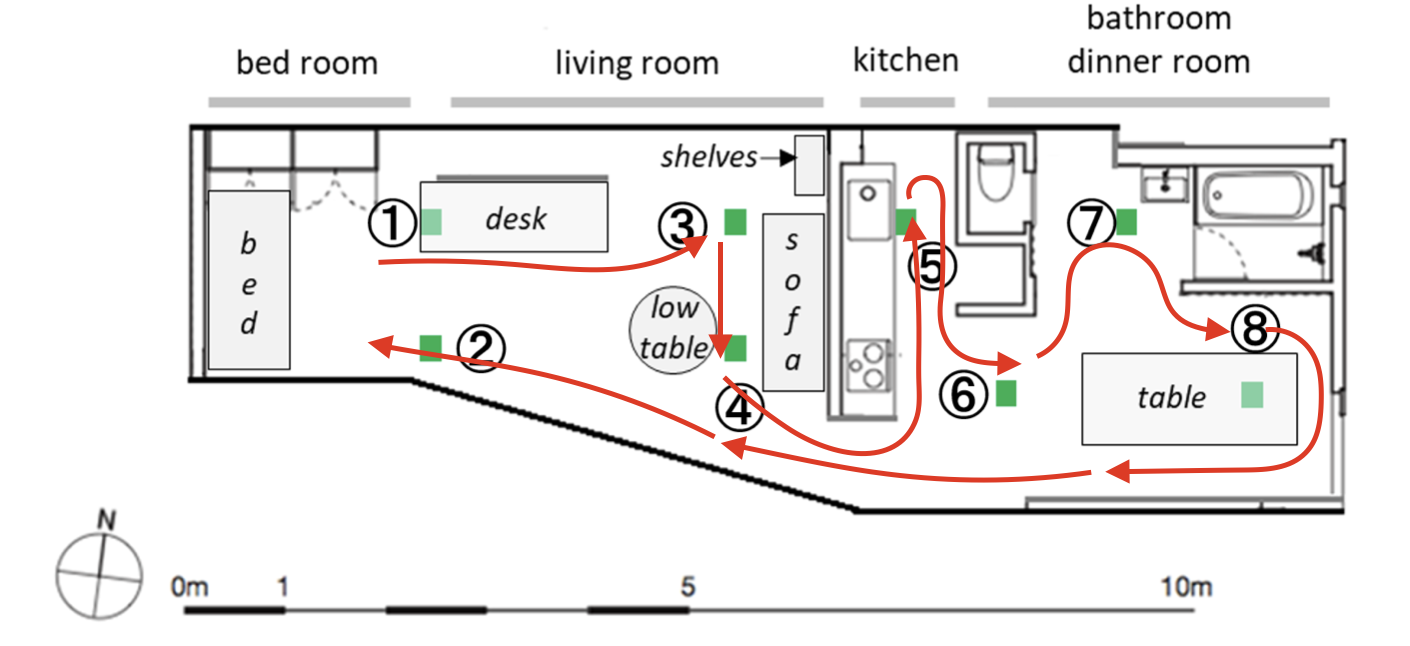


**Figure 6. Walking trial path.**

For each participant, the two following data were taken from this trial:

- The gait type of each participant (heel or midfoot landing) was visually determined using the video records. The evaluation was conducted by two researchers specializing in gait analysis.
- The average landing impact force of each participant was calculated by averaging the peak values found in the uniformized vector norm time series that correspond to all steps performed during the walking trial. This parameter was divided by three to obtain the criterion value used for the extraction of the *moving distance* parameter.

**References**

1. Kitayama Asami. Development of a method to estimate physical activity using floor vibration information: development of floor vibration calibration procedure to be apply for general housing. Master thesis, Joint Program in Life Science, 2022. [Original title: 北山亜紗美. 床振動情報を利用した身体活動量推定モデルの開発 〜一般住宅に適用するための床振動較正法の確立〜. 生活工学共同専攻,修士論文, 2022年度.]
2. Hiroyuki Harada, Hideyuki Kosaka (2008). Fundamental Study of Exciting Force Used for the Estimation of the Severity of a Floor Vibration - Estimation of Human Induced Exciting Force and Decision of Standard Impulsive Force - *Reports of Technical Research Institute of Sumitomo Mitsui Construction Co., Ltd. (CD-ROM)*
