# Supplementary material for: Objective Assessment of Physical Activity at Home Using a Novel Floor-Vibration Monitoring System: Validation and Comparison With Wearable Activity Trackers and Indirect Calorimetry Measurements
Source: JMIR Form Res. 2024 Apr 25;8:e51874. doi: 10.2196/51874 (PMC11082727; doi:10.2196/51874)
Supplement: Multimedia Appendix 2 [file formative_v8i1e51874_app2.docx]

**Additional results**

**Moving distance parameter**

The *moving-distance* parameter computed from the floor vibration signal was compared to the distances estimated from the observations of video records. The statistical analysis reveals a high correlation between the both methods (r = 0.88, p<0.001, spearman).

**
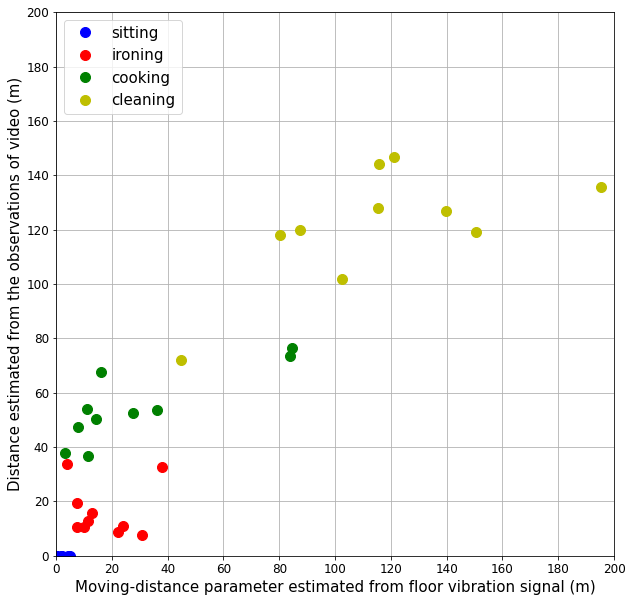
**

**Figure 1. Relationship between the *moving-distance* parameter and distances estimated from the observations of video.**

**Waist- and wrist-worn activity trackers**

Figure 2 and Figure 3 below show the ANOVA results for parameters that were not included in the Figure 4 of the main body of the manuscript.


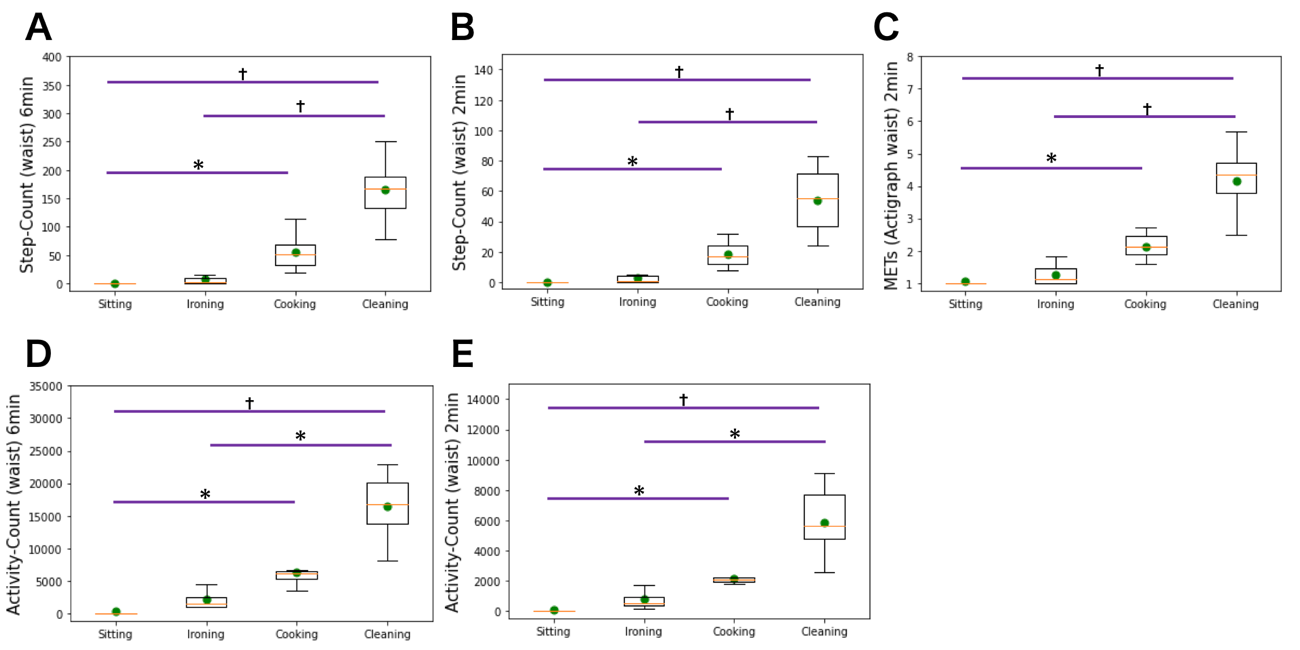


**Figure 2. Comparison between the four experimental home activities for the waist-worn Actigraph GT9X device.** (A) step-count obtained over the 6 minutes; (B) step-count obtained over the last 2 minutes corresponding to the Douglas bag measurements; (C) Activity intensities computed from data obtained over the last 2 minutes corresponding to the Douglas bag measurements (“Crouter adult (2010)” equation); (D) *activity-count* vector magnitudes obtained over the 6 minutes; (E) *activity-count* obtained over the last 2 minutes corresponding to the Douglas bag measurements. The *activity-count* parameter depends upon the amplitude and frequency of the acceleration events over an adjustable time window, which is also called an epoch. The *activity-count* parameter is a three-dimensional parameter. While the *activity-count* vector magnitude is sometimes used as a quantitative index of the physical activity, this parameter can also be processed further to obtain the energy expenditure or activity intensity predictions expressed in kilocalories or metabolic equivalent of task (MET), respectively [1]. The intensity predictions of the GT9X monitors were computed using the “Crouter adult (2010)” equation [2]. Yellow line: median. Green point: average. Outliers are not depicted. *: *P*<.05, †: *P*<.001.


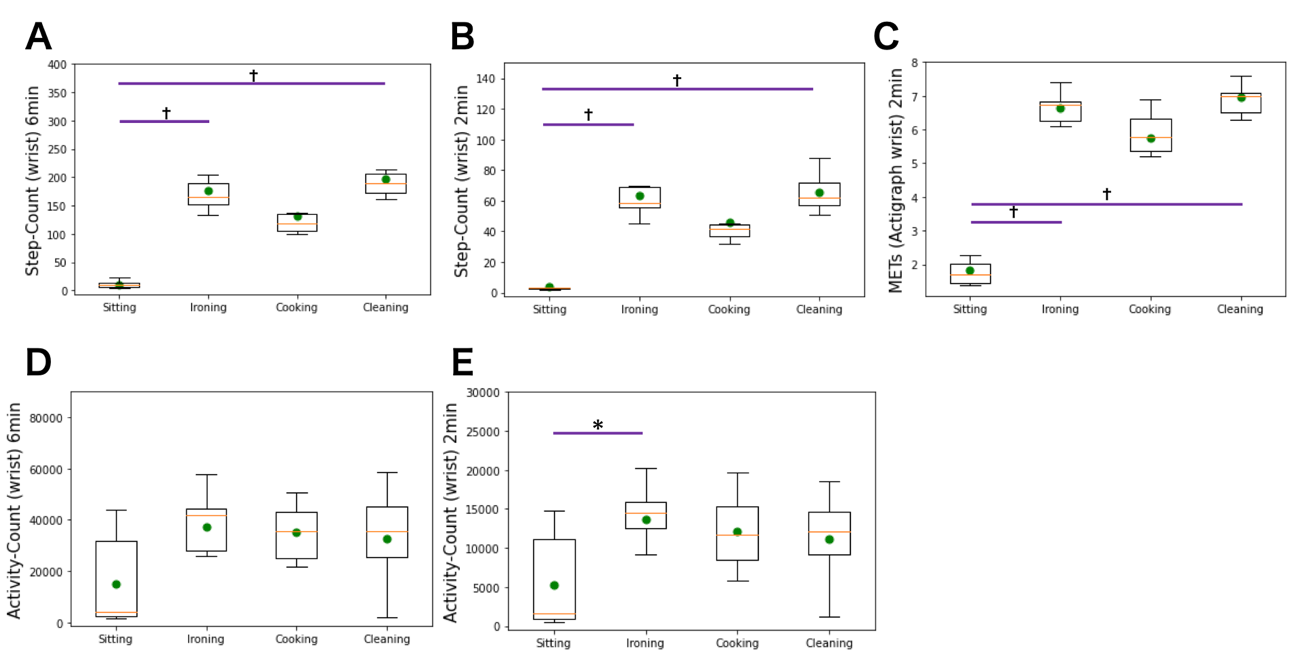


**Figure 3. Comparison between the four experimental home activities for the wrist-worn Actigraph GT9X device.** (A) step-count obtained over the 6 minutes; (B) step-count obtained over the last 2 minutes corresponding to the Douglas bag measurements; (C) Activity intensities computed from data obtained over the last 2 minutes corresponding to the Douglas bag measurements (“Crouter adult (2010)” equation); (D) *activity-count* vector magnitudes obtained over the 6 minutes; (E) *activity-count* obtained over the last 2 minutes corresponding to the Douglas bag measurements. The *activity-count* parameter depends upon the amplitude and frequency of the acceleration events over an adjustable time window, which is also called an epoch. The *activity-count* parameter is a three-dimensional parameter. While the *activity-count* vector magnitude is sometimes used as a quantitative index of the physical activity, this parameter can also be processed further to obtain the energy expenditure or activity intensity predictions expressed in kilocalories or metabolic equivalent of task (MET), respectively [1]. The intensity predictions of the GT9X monitors were computed using the “Crouter adult (2010)” equation [2]. Yellow line: median. Green point: average. Outliers are not depicted. *: *P*<.05, †: *P*<.001.

**Multiple regressions: additional models**

The Table 1 presented alternative models (forced entry multiple linear regression). The models 6 and 7 associate *moving-distance*, which integrates the spatial dimension of the motion, with either *floor-count* or *step-count*, which focus on the physical dimension. Model 8 associate the four objective parameters only. Considering future applications, it may indeed be challenging for people to proceed to a self-assessment of their own gait. In addition, gait may change over time. Entering this gait type information in the system to use it as a predictor of EE may therefore be challenging. Assuming that the *floor-count* parameter could be related to body weight (see Table 2 below), Model 9 has been built with *step-count*, *moving-distance*, body weight but not *floor-count*. Similar considerations on the possible relationship between *floor-count*, body weight and also gait type led to the development of the model 10. Interestingly, the models 7-10 allows explaining the variation in activity intensities by 82 to 87%.

Stepwise multiple regression procedures have also been performed. The outcomes are similar to model 2 and 4 for α-to-enter/to-remove values of 0.15 and 0.30, respectively.

**Table 1.** Relationship between floor vibration-based parameters and actual activity intensities evaluated [additional models]

| Models | Predictor variables | SPRC | *P-*value | r^2^ |
| --- | --- | --- | --- | --- |
| Multiple regressions |  |  |  |  |
| 6 | *moving-distance*  *floor-count* | 0.726  0.100 | 0.0012  0.63 | 0.67 |
| 7 | *step-count*  *moving-distance* | 0.781  0.143 | < 0.001  0.30 | 0.82 |
| 8 | *floor-count*  *step-count*  *moving-distance*  body weight | -0.381  0.953  0.334  -0.067 | 0.03  < 0.001  0.04  0.32 | 0.86 |
| 9 | *step-count*  *moving-distance*  body weight | 0.787  0.140  -0.111 | < 0.001  0.30  0.11 | 0.83 |
| 10 | *step-count*  *moving-distance*  body weight  gait type | 0.826  0.146  -0.106  -0.190 | < 0.001  0.23  0.09  0.005 | 0.87 |

Models 1 to 5 are shown in the main document (Table 2 of the main article). SPRC: Standardized Partial Regression Coefficient.

**Statistical relationship between predictors**

**Table 2.** Correlation (spearman) between all parameters used in the multiple regression models.

| *step-count* | r=0.97  *P*<.001 |  |  |  |
| --- | --- | --- | --- | --- |
| *moving-distance* | r=0.87  *P*<.001 | r=0.83  *P*<.001 |  |  |
| body weight | r=0.03  *P*=0.84 | r=0.01  *P*=0.949 | r=-0.031  *P*=0.851) |  |
| gait type | r=0.198  *P*=0.220 | r=0.139  *P*=0.391 | r=0.066  *P*=0.684 | r=0.076  *P*=0.640 |
|  | *floor-count* | *step-count* | *moving-distance* | body weight |

**References**

1. Chen KY, Bassett DR Jr. The technology of accelerometry-based activity monitors: current and future. Med Sci Sports Exerc. 2005;37(11 Suppl):S490-500. doi:10.1249/01.mss.0000185571.49104.82.
2. Crouter SE, Kuffel E, Haas JD, Frongillo EA, Bassett DR Jr. Refined two-regression model for the ActiGraph accelerometer. Med Sci Sports Exerc 2010;42(5):1029-1037. doi:10.1249/MSS.0b013e3181c37458.
